# Isolation and Characterization of Gut Bacterial Proteases Involved in Inducing Pathogenicity of *Bacillus thuringiensis* Toxin in Cotton Bollworm, *Helicoverpa armigera*

**DOI:** 10.3389/fmicb.2016.01567

**Published:** 2016-10-06

**Authors:** Visweshwar Regode, Sreeramulu Kuruba, Akbar S. Mohammad, Hari C. Sharma

**Affiliations:** ^1^Department of Entomology, International Crops Research Institute for the Semi-Arid Tropics, PatancheruIndia; ^2^Department of Biochemistry, Gulbarga University, KalaburagiIndia

**Keywords:** *Helicoverpa armigera*, midgut bacteria, proteases, Cry1Ac proteins, transgenics

## Abstract

*Bacillus thuringiensis* toxin proteins are deployed in transgenic plants for pest management. The present studies were aimed at characterization of gut bacterial proteases involved in activation of inactive Cry1Ac protoxin (pro-Cry1Ac) to active toxin in *Helicoverpa armigera.* Bacterial strains were isolated from *H. armigera* midgut and screened for their proteolytic activation toward pro-Cry1Ac. Among 12 gut bacterial isolates seven isolates showed proteolytic activity, and proteases from three isolates (IVS1, IVS2, and IVS3) were found to be involved in the proteolytic conversion of pro-Cry1Ac into active toxin. The proteases from IVS1, IVS2, and IVS3 isolates were purified to 11.90-, 15.50-, and 17.20-fold, respectively. The optimum pH and temperature for gut bacterial protease activity was 8.0 and 40°C. Maximum inhibition of total proteolytic activity was exerted by phenylmethane sulfonyl fluoride followed by EDTA. Fluorescence zymography revealed that proteases from IVS1, IVS2, and IVS3 were chymotrypsin-like and showing protease band at ~15, 65, and 15 kDa, respectively. Active Cry1Ac formed from processing pro-Cry1Ac by gut bacterial proteases exhibited toxicity toward *H. armigera*. The gut bacterial isolates IVS1, IVS2, and IVS3 showed homology with *B. thuringiensis* (CP003763.1), *Vibrio fischeri* (CP000020.2), and *Escherichia coli* (CP011342.1), respectively. Proteases produced by midgut bacteria are involved in proteolytic processing of *B. thuringiensis* protoxin and play a major role in inducing pathogenicity of *B. thuringiensis* toxins in *H. armigera*.

## Introduction

Cotton bollworm, *Helicoverpa armigera* (Hubner) (Lepidoptera: Noctuidae) is polyphagous pest. It is distributed all over the world, and feeds on more than 250 plant species, including the agriculturally important crops cotton, tomato, sunflower, corn, pigeonpea, chickpea, and vegetable and field crops (Sharma, 2005). Overdependence on insecticide use has not only resulted in development of insect resistance to insecticides, but also leaves harmful residues on food products. Therefore, plants expressing toxin genes produced from the bacteria *Bacillus thuringiensis* have been deployed on a large scale for controlling insect pests (Sharma et al., 2004; James, 2013).

*Bacillus thuringiensis* is an aerobic, gram-positive, spore-forming bacterium that produces delta-endotoxins (Schnepf et al., 1998; De Maagd et al., 2001). The *B. thuringiensis* formulation sprays have been widely used as a biopesticide for controlling the insect pests (Sanahuja et al., 2011). After ingestion by the larvae, the *B. thuringiensis* protoxin undergo proteolysis in midgut under the alkaline environment, the active toxin formed will binds to receptors on the midgut epithelium, and then active toxin inserted into the membrane, creates membrane pores and eventually results in cell lysis leading to death of insect (Grochulski et al., 1995; Aronson and Shai, 2001). Pest insects had gained resistance to *B. thuringiensis* toxins and the cause of resistance may be due to lack of major gut protease involved in protoxin cleavage (Oppert et al., 1997), variations in the expression pattern of midgut proteases (Keller et al., 1996; Karumbaiah et al., 2007), improper processing of protoxin by proteases (Li et al., 2004; Rajagopal et al., 2009), and reduced binding of the active toxin to the receptors on midgut epithelium (Wang et al., 2007; Nair et al., 2013). Resistance to *B. thuringiensis* toxins is also associated with changes in expression of receptors, glycosylphosphatidyl-inositol (GPI) anchored alkaline phosphatases (ALPs) (Fuentes et al., 2011), GPI anchored aminopeptidases-N (APN) (Tiewsiri and Wang, 2011), cadherin (CAD) (Fabrick et al., 2014), and ABC transporter loci (Baxter et al., 2011).

Some insects had gained resistance to *B. thuringiensis* in the absence of midgut bacteria. Midgut bacteria are required for *B. thuringiensis* pathogenicity, and there is an obligate association between *B. thuringiensis* subsp. *kurstaki* HD-1 and midgut microbiota in some insect species [e.g., *Lymantria dispar* (L.), *Pieris rapae* (L.), and *Vanessa cardui* (L.)] (Broderick et al., 2009). Elimination of gut bacteria by oral administration of antibiotics reduced the *B. thuringiensis* insecticidal activity in gypsy moth (Broderick et al., 2006) and in *H. armigera* (Paramasiva et al., 2014; Visweshwar et al., 2015), however, the *B. thuringiensis* toxicity is restored by inoculation of a gut-associated strain of *Enterobacter* in gypsy moth (Broderick et al., 2006). There are reports that midgut bacteria are not necessary for *B. thuringiensis* insecticidal activity in pink bollworm, *Pectinophora gossypiella* (Saunders) (Broderick et al., 2009) and *Plutella xylostella* (L.) (Raymond et al., 2009).

In earlier studies (Visweshwar et al., 2015), we found that the insecticidal activity of *B. thuringiensis* toxins was reduced in *H. armigera* larvae eliminated with gut bacteria using antibiotic cocktail. The present studies were therefore carried out to know whether the proteases produced from *H. armigera* gut bacteria are involved in proteolytic processing of pro-Cry1Ac to active toxin and also to understand the role of gut bacterial proteases in inducing Cry1Ac toxicity in *H. armigera*.

## Materials and Methods

### Insect Culture

The *H. armigera* larvae were reared on chickpea based artificial diet under controlled laboratory conditions [Temperature at 26 ± 1°C, 60–70% relative humidity, and photoperiod of 16:8 h (L:D)] (Chitti Babu et al., 2014) in the insect rearing laboratory at the International Crops Research Institute for the Semi-Arid Tropics (ICRISAT), Patancheru, Telangana, India.

### Isolation of Gut Bacteria form *H. armigera* Larvae

The neonates were reared on artificial diet till they attain fourth-instar. The healthy early fourth-instar larvae were kept for starvation for 3 h, then surface sterilized with distilled water fallowed by 70% ethanol. Larvae were transferred to Petri plates containing paraffin, immobilized with surgical pins, covered with sterile water, and dissected using surgical blade. All the dissecting instruments were autoclaved before use. Only midgut portions were collected in sterile 0.1 M phosphate buffer, pH 7.0, homogenized and serially diluted aseptically. Different dilutions (10^-3^, 10^-5^, and 10^-7^) were inoculated on half strength tryptic soya-agar (TSA) media by spread plate method. The media was incubated at 30°C for 48 h. Total 10 larvae were used for isolation of midgut and all the steps were performed under sterile conditions in laminar air flow hood.

### Determination of Proteolytic Activity in the Bacterial Culture

To determine the bacterial proteolytic activity, single bacterial colony on TSA media was selected and inoculated on skim milk agar media (0.5% skim milk and 1.5% agar). The inoculated media was incubated at 30°C for 24 h. The bacteria with proteolytic activity formed a clear zone around the inoculum. Protease producing bacterial colonies were picked for further characterization.

### Production of Proteases from Bacterial Culture

The midgut bacteria showing proteolytic activity were selected and screened for the protease production on Glucose-Yeast-Peptone (GYP) media (Kumar et al., 2002) [glucose (10 g/l), yeast extract (5 g/l), peptone (5 g/l), MgSO_4_ 7H_2_O (0.2 g/l), K_2_HPO_4_ (1 g/l), pH 9.5]. GYP broth was inoculated with 10% (v/v) of 24 h old seed culture prepared in nutrient broth and the culture was incubated at 30°C for 48 h. The culture was centrifuged at 10,000 rpm for 15 min, pellet was discarded and supernatant used as protease source.

### Gut Bacterial Protease Activity Assay

Total protease activity in gut bacteria was determined by using azocasein (Sigma-Aldrich, India) as a substrate (Vinod et al., 2010; Visweshwar et al., 2015). The culture extract (50 μl) was mixed with the 500 μl substrate (1% azocasein in 0.1 M glycine-NaOH buffer, pH 8.0) and incubated for 50 min. Then 200 μl of 5% trichloroaceticacid (TCA) was added and centrifuged at 10000 rpm for 10 min at 25°C. To the supernatant, equal volume of 1 N NaOH was added and absorbance recorded at 450 nm. Units for total protease activity (UA) were calculated by using the equation units activity (UA) = ABS_450__nm_/[time (min) x volume of enzyme (ml)].

### Purification of Gut Bacterial Proteases

The midgut bacterial culture supernatant was partially purified by salt precipitation. Ammonium sulfate was dissolved slowly in culture supernatant to achieve 75% saturation. After 16 h, the precipitate was recovered by centrifugation at 10,000 × *g* for 20 min. The precipitate was dissolved in 10 mM Tris-HCl buffer (pH 8.0), and dialyzed overnight against the same buffer. The dialyzed enzyme preparation was applied on diethylaminoethyl-cellulose (DEAE-cellulose) (Sigma-Aldrich, India) column (50 cm × 2 cm) pre-equilibrated with 10 mM Tris-HCl (pH 8.0) and un-adsorbed protein fractions were eluted with the same buffer. The enzyme was eluted with a gradient of 0.1–1 M NaCl in the same buffer at a flow rate of 1 ml/min. In each eluted fraction, total protease activity was determined using azocasein as substrate. The active fractions containing protease activity were pooled, concentrated by Amicon^®^ Pro Affinity Concentrator (Millipore, USA) and then loaded on Sephadex G-75 (Sigma-Aldrich, India) column (90 cm × 2 cm), previously equilibrated with 0.2 M phosphate buffer (pH 7.5) and developed at a flow rate of 0.5 ml/min. Five milliliter fractions were eluted with the same buffer and fractions containing protease activity were pooled, concentrated to 2 ml by Amicon^®^ Pro Affinity Concentrator. The protein concentration was quantified using bovine serum albumin (BSA) as standard protein (Lowry et al., 1951).

### Bacterial Proteases in Protoxin–Toxin Conversion

Pro-Cry1Ac was isolated by fermenting *B. thuringiensis* subsp. *kurstaki* 4D4 strain (supplied by Daniel R. Zeigler, Bacillus Genetic Stock Center, Ohio State University, Columbus, OH, USA) at 30°C in Luria-Bertaini (LB) medium (Shao et al., 1998). After 48 h, the fermented medium was collected and kept at 4°C until cells lysis. The spore and crystal mixture were precipitated by centrifugation at 10,000 *g* × 10 min, the resultant pellet was washed three times with 1 M NaCl to remove the endogenous proteases, followed with distilled water. Delta-endotoxins were selectively dissolved in 2% β-mercaptoethanol-NaOH buffer, pH 10.7, and centrifuged at 10,000 *g* × 20 min under 4°C. Pro-Cry1Ac was dissolved in the supernatant. Acetic acid (2 mM) was added to the supernatant to adjust pH 4.4, and then centrifuged at 10,000 *g* for 30 min. The precipitate of pro-Cry1Ac was collected, dialyzed against water, lyophilized, and stored at -20°C.

Gut bacterial proteases were incubated with pro-Cry1Ac for 1 h at room temperature and the samples were electrophoresed on sodium dodecyl polyacrylamide gel electrophoresis (SDS-PAGE) using 10% (w/v) running gel (Laemmli, 1970) under reducing conditions. The same samples were subjected to ELISA using *B. thuringiensis*-Cry1Ac ELISA kit (Agdia^®^, India) for quantification of active Cry1Ac. For immunodetection, the proteins were separated by SDS-PAGE as described above and electroblotted onto a nitrocellulose membrane in electrophoretic transfer buffer (48 mM Tris base, 39 mM glycine, 20% methanol and 1.3 mM SDS, pH 9.2). The membrane was blocked in PBST (155 mM NaCl, 1.1 mM KH_2_PO_4_, 3.0 mM K_2_HPO_4_ 7H_2_O, pH 7.4, 0.25% Tween-20) plus 5% skimmed milk for 1 h at room temperature and then incubated with primary antibody in PBST overnight at 4°C. The primary antibody used for Western blot analyses was monoclonal mouse anti-Cry1Ac antibody [1:1000 (v/v); RDI, USA]. The membranes were then washed three times with PBST, followed by incubation with horseradish peroxidase-conjugated secondary antibody [1:4000 (v/v); Agdia^®^, India] in PBST. The immunoreactivity was detected by incubating the membrane in PBS containing 0.06% (w/v) diaminobenzidine, 0.018% NiCl_2_ (w/v), and 0.3% H_2_O_2_ (v/v).

### Effect of pH and Temperature on the Activity of Gut Bacterial Proteases

Effect of pH on gut bacterial proteases was determined by incubating the proteases with substrate azocasein at varying pH values ranging from 2.0 to 12.0, with intervals of 2 units using 100 mM aconitate buffer for pH 2.0–5.0, 100 mM sodium phosphate buffer for pH 6.0–7.0 and 100 mM glycine-NaOH for pH 8.0–12.0. The influence of temperature on gut bacterial protease was studied by incubating the proteases with the substrate azocasein at different temperatures ranging from 20 to 70°C, with intervals of 10°C for 10 min under the standard assay conditions. The total proteolytic activity was determined as described above.

### Effect of Inhibitors on Protease Activity

For inhibitory assays, a suitable amount of inhibitor (5 mM) was pre-incubated with *H. armigera* gut bacterial protease extract (50 μl) for 30 min at room temperature. After incubation, total protease activity was determined by using azocasein as a substrate. Chymotrypsin activity was estimated by using substrate *N*-succinyl-alanine-alanine-proline-phenylalanine-*p*-nitroanilide (SAAPFpNA) (Sigma-Aldrich, India) (Li et al., 2004; Visweshwar et al., 2015). One unit of enzyme activity was defined as the amount of enzyme catalyzing the hydrolysis of 1 μmol substrate per minute at 30°C. Protease inhibitors used were TPCK (*N*-tosyl-L-phenylalanine chloromethyl ketone), E64 [*N*-(*N*-(*L*-3*-trans*-corboxirane-2-carbonyl)-L-leucyl) agmatine], leupeptin, aprotonin, EDTA (ethylene diamine tetra acetic acid), and PMSF (phenylmethane sulfonyl fluoride). Residual protease activity in presence of inhibitors was presented as relative to activity in control (activity in the absence of protease inhibitor). All the protease inhibitors were purchased from Roche Diagnostics, Mumbai, India.

### Fluorescence Zymogram Analysis of Midgut Bacterial Proteases

The gut bacterial proteases were subjected to 10% SDS-PAGE under non-reducing conditions. After electrophoresis, the gel was treated with 2.5% triton X-100 for 10 min to remove the SDS. The gel was incubated in trypsin assay buffer [50 mM Tris-HCl, pH 8.0, 10 mM CaCl_2_, 0.005% Triton X-100 and 50 μM *N*-tert-butoxycarbonyl-glutamine-alanine-arginine-7-amido-4-methyl coumarin (Boc-Gln-Ala-Arg-MCA)] (Sigma-Aldrich, India) or chymotrypsin assay buffer [50 mM Tris-HCl, pH 8.0, 10 mM CaCl_2_, 0.005% Triton X-100 and 50 μM *N*-succinyl-alanine-alanine-proline-phenylalanine-7-amido-4-methyl coumarin (Suc-Ala-Ala-Pro-Phe-MCA)] (Sigma-Aldrich, India) at 37°C for 30 min. The gel was then washed in distilled water and observed for fluorescent bands of trypsin and chymotrypsin activity in Gel Documentation System under UV-B transillumination (Bio-Rad Laboratories, Hercules, CA, USA) (385 nm = AMC group excitation wavelength) (Yasothornsrikul and Hook, 2000). In one lane, standard molecular weight markers were loaded to determine the molecular mass of the purified gut bacterial proteases. After electrophoresis, the lane was sliced and stained with Coomassie brilliant blue (R-250) and then de-stained.

### Preparation of Active Cry1Ac Toxin

The purified crystal δ-endotoxin was solubilized in 0.1 M carbonate/dithiothreitol buffer containing 1 M sodium chloride and incubated with proteases (5:1 v/v) from midgut bacteria at 30°C for 60 min. Pro-Cry1Ac digestion was stopped with 1 mM PMSF and the samples were centrifuged at 16,000 *g* for 10 min. The toxin-containing supernatant was harvested and protein concentration was determined by the method of Lowry et al. (1951).

### Elimination of Gut Bacteria from *H. armigera*

To eliminate the gut bacteria, eggs laid by *H. armigera* adults were surface sterilized by immersing in distilled water followed by 2% sodium hypochlorite and then allowed to hatch. Stock solutions of antibiotic cocktail were prepared using antibiotics, gentamycin, neomycin, chloramphenicol, ampicillin, streptomycin, rifampicin, and penicillin (Sigma-Aldrich, India) (stock: 0, 0.2, 0.4, 0.6, 0.8, 1.0 mg/ml each in distilled water). The neonates were released on sterilized artificial diet treated with 100 μl of antibiotic cocktail (from each stock solution) per gram of diet and the larvae were reared until they reach fourth-instar. The early fourth-instar larva was surface sterilized, midguts (10 no.) were removed aseptically and homogenized in sterile distilled water. The gut homogenates were serially diluted and spread on nutrient agar (NA) media and incubated at 30°C for 24 h. The microbial counts were converted into colony forming units per mg of whole gut. There were three replications for each treatment and 10 larvae in each replication in a completely randomized design (CRD).

### Insect Bioassay

For insect bioassay, pro-Cry1Ac and Cry1Ac toxin produced after proteolytic processing of pro-Cry1Ac by three gut bacterial (IVS1, IVS2, and IVS3) proteases were used. Fifty microliters of pro-Cry1Ac and active Cry1Ac toxin (0.1, 0.3, 0.6, 1.2, 2.4, 4.8, and 9.6 μg) samples were coated uniformly to the surface (2 cm^2^) of an artificial diet (antibiotic cocktail untreated) and also to artificial diet pre-treated with 100 μl of antibiotic cocktail per gram diet (antibiotic cocktail stock: 1 mg/ml of each antibiotic) and allowed to dry. Each early fourth-instar larva reared on artificial diet alone and antibiotic cocktail treated diet was placed onto the surface of diet treated with toxin and diet treated with antibiotic cocktail plus toxin, respectively. The larvae were reared under optimal conditions (see Insect Culture). Insect mortality was scored after 5 days. There were three replications for each treatment and 10 larvae in each replication in a CRD. The LC_50_ values (effective concentration to kill 50% of the *H. armigera* larvae) were calculated using probit analysis (LC50 software program, version 1.5 developed by EPA; Finney, 1971).

To test whether midgut bacteria were contributing to Cry1Ac induced mortality directly, the early fourth-instar larvae pre-treated with antibiotic cocktail were transferred to diet treated with both pro-Cry1Ac and gut bacterial culture. There were three replications for each treatment and 10 larvae in each replication in a CRD. The mortality data was recorded after 5 days.

### MALDI-TOF Mass Spectrometry

The selected protease band from IVS1, IVS2, and IVS3 isolates were excised from the Coomassie Blue stained gel, and washed thrice with water for 10 min, and stored in milli Q water. MALDI time-of-flight mass spectrometry (TOFMS) analysis of proteases were performed using a MALDI-tandem time-of-flight (TOF/TOF) mass spectrometer (Bruker Autoflex III Smartbeam; Bruker Daltonics, Bremen, Germany). Protein identification was done by database searches (PMF and MS/MS) using MASCOT program^1^ employing Biotools software (Bruker Daltonics) (Himabindu et al., 2013). The similarity search for mass values was done with existing digests and sequence information from NCBInr and Swiss Prot database. The other search parameters were: fixed modification of carbamidomethyl (C), variable modification of oxidation (M), enzyme trypsin, peptide charge of 1^+^ and monoisotropic. According to the MASCOT probability analysis (*P* < 0.05), only significant hits were accepted for protein identification. The multiple alignment was made using Clustal Omega software.

### Characterization of Gut Protease Producing Bacterial Strains

The gut bacterial strain, involved in conversion of protoxin–toxin was characterized by biochemical and molecular methods. For biochemical characterization of the proteolytic strain, tests such as nitrate reduction, citrate utilization, H_2_S production, catalase, gelatin liquification, starch hydrolysis, and urease test were performed as per standard protocols (Hemaraj et al., 2013). Results were analyzed as per Bergey’s Manual of Systematic Bacteriology (Holding and Shewan, 1974).

Molecular characterization was carried out using 16S rRNA gene sequence analysis. DNA was isolated from a single bacterial colony using Biopure^TM^ kits (Bioaxis DNA Research Centre, Hyderabad, India) for bacterial genomic DNA isolation. 16S rRNA gene was amplified using 16S universal primers (forward, AGAGTTTGATCCTGGCTCAG; reverse, ACGGCTACCTTGTTACGACTT). Forward and reverse sequencing reaction of PCR amplicon was carried out on ABI PRISM^®^ 377 Genetic Analyzer to obtain the sequence. The sequences were deposited in the GenBank. Related sequences were obtained from the GenBank database (National Center for Biotechnology information) using BLAST. A phylogenetic tree was constructed by the neighbor-joining method using the MEGA 7.0 software (Kumar et al., 2016).

## Results

### Purification and Activity of Proteases Isolated from Midgut Bacteria of *H. armigera*

**FIGURE 1:**
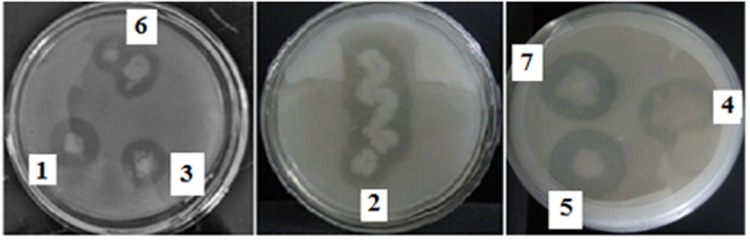
**Screening of *Helicoverpa armigera* midgut bacterial isolates for protease activity.** Gut bacterial isolates were inoculated on agar plate containing skimmed milk casein and incubated for 24 h at 30°C. The clear zone around the inoculum indicated the hydrolysis of skimmed milk casein by the proteases secreted by gut bacteria.

Twelve dominant microbial colonies were isolated from the midgut of *H. armigera*. Amongst these, only seven gut bacterial isolates exhibited protease activity, as indicated by formation of a clear zone around the inoculum on skim milk agar media, and these isolates were named as IVS-1 to 7 (**Figure 1**). The midgut bacterial proteases were salt precipitated and were further purified by ion-exchange and gel permeation chromatography (**Table 1**). Proteases from IVS1 isolate were purified to 27.19% yield with 11.90-fold increase in activity, and the specific activity was 6.14 UA/mg of protein. Proteases from IVS2 isolate were purified to 49.43% yield with 15.50-fold increase in activity. Specific activity of protease was 6.34 UA/mg of protein. Proteases from IVS3 isolate were purified to 29.48% yield with 17.20-fold increment in activity, and the specific activity was 8.22 UA/mg of protein (data has been provided for only three isolates of gut bacteria which were able to convert pro-Cry1Ac to active toxin).

**Table 1:** Midgut bacterial protease activities.

Purification stage	Volume (ml)	Protein (mg/ml)	Total activity (UA/ml)	Specific activity (UA/mg of protein)	Purification fold	%Yield
**IVS1**						
Crude	1000	1.883	0.971	0.516	1.0	100
Salt precipitation	10	0.602	0.6074	1.009	2.0	62.55
DEAE-cellulose	34	0.111	0.398	3.586	7.0	40.99
Sephadex G75	22	0.043	0.264	6.140	11.9	27.19
**IVS2**						
Crude	1000	1.728	0.706	0.409	1.0	100
Salt precipitation	10	0.556	0.548	0.986	2.4	77.62
DEAE-cellulose	36	0.101	0.415	4.109	10.1	58.78
Sephadex G75	24	0.055	0.349	6.345	15.5	49.43
**IVS3**						
Crude	1000	1.811	0.865	0.478	1.0	100
Salt precipitation	10	0.45	0.664	1.476	3.1	76.76
DEAE-cellulose	36	0.098	0.443	4.520	9.5	51.21
Sephadex G75	18	0.031	0.255	8.226	17.2	29.48

*Each value represents the average of three experiments.*

**FIGURE 2:**
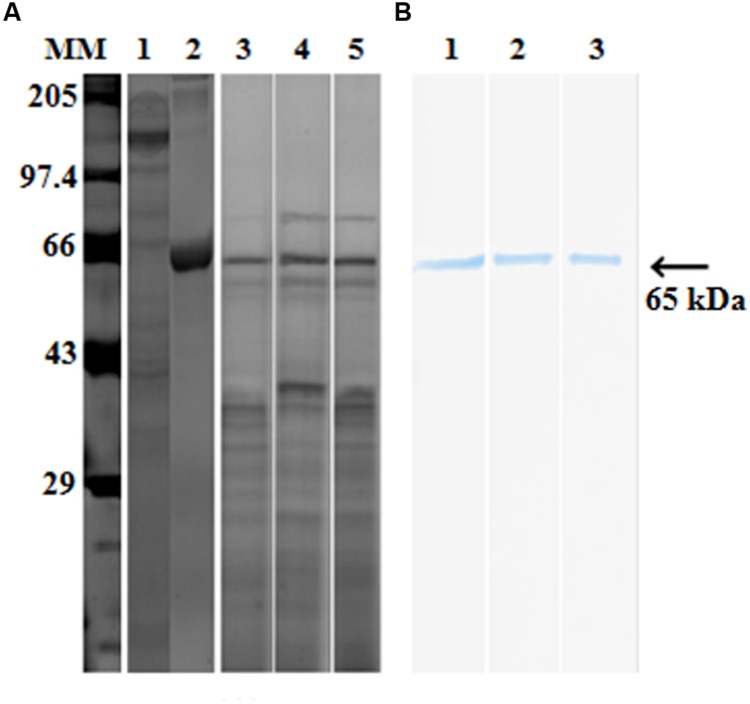
**Proteolytic processing of pro-Cry1Ac to active toxin by midgut bacterial proteases.** Proteases (30 μg) from midgut bacteria were incubated with pro-Cry1Ac (10 μg) for 1 h. Active Cry1Ac (band at 65 kDa) was detected on **(A)** SDS-PAGE [molecular markers (MMs), lane 1- pro-Cry1Ac, lane 2- active Cry1Ac, lane 3 – IVS1, lane 4 – IVS2 and lane 5 – IVS3] and **(B)** Western blot (lane 1- IVS1, lane 2- IVS2, and lane 3- IVS3).

Purified proteases from seven protease producing gut bacterial isolates were incubated with pro-Cry1Ac for 1 h at room temperature. Proteases from only three isolates (IVS1, IVS2, and IVS3) were involved in proteolytic processing of pro-Cry1Ac which showed a band corresponding to active Cry1Ac toxin at 65 kDa on SDS-PAGE (**Figure 2A**), while remaining four isolates (IVS4, IVS5, IVS6, and IVS7) did not show any band correspond to 65 kDa. This was also confirmed by detecting the proteolytic samples for the presence of active toxin using ELISA (**Table 2**) and Western blot (**Figure 2B**).

**Table 2:** Quantification of active toxin produced from pro-Cry1Ac by gut bacterial proteases.

Isolate	Cry1Ac (μg)/mg protein
IVS1	0.20 ± 0.03
IVS2	0.12 ± 0.01
IVS3	0.15 ± 0.02
IVS4	–
IVS5	–
IVS6	–
IVS7	–

*Bacterial proteases (50 μg) incubated with pro-Cry1Ac for 1 h. Sample (100 μl) was added to ELISA plate wells and the reaction was carried out as described in M&Ms. The values were represented as mean ± SE.*

Proteolytic gut bacterial isolates IVS1, IVS2, and IVS3 were characterized by biochemical and molecular methods. Microscopic observations showed that all the three bacterial isolates were rod shaped. Biochemical tests confirmed that all the three isolates showed negative results for H_2_S production, urease test, and citrate utilization test (**Table 3**). Based on the biochemical tests and 16S rRNA gene sequence analysis the three isolates, IVS1, IVS2, and IVS3 were identified as *B. thuringiensis* HD-789 (CP003763.1; 99% similarity), *Vibrio fischeri* ES114 (CP000020.2; 95% similarity) and *Escherichia coli* GM-4792 (CP011342.1; 99% similarity), respectively. The sequences have been deposited in GenBank under the accession numbers KT714122 (IVS1), KT714123 (IVS2), and KT714124 (IVS3) (The phylogenetic tree for IVS1, IVS2, and IVS3 isolates was provided as Supplementary Figure S1).

**Table 3:** Morphological and biochemical characterization of proteolytic bacterial isolates from *Helicoverpa armigera*.

Bacterial isolate	Shape	Gram staining	H_2_S	Urease	Citrate	Catalase	Starch	Gelatin	Nitrate
IVS1	Rod	+	-	-	-	+	+	+	+
IVS2	Rod	-	-	-	-	+	+	+	-
IVS3	Rod	-	-	-	-	+	-	-	+

### Effect of pH and Temperature on Gut Bacterial Protease Activity

**FIGURE 3:**
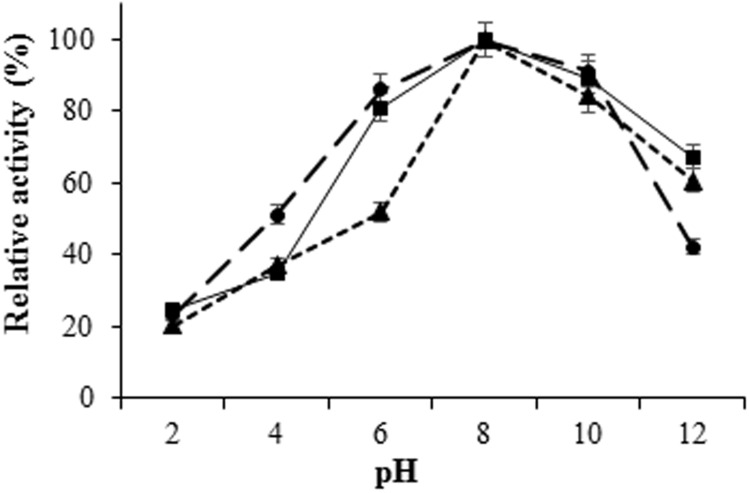
**Effect of pH on activity of proteases from gut bacteria in *H. armigera*.** The midgut bacterial proteases were incubated with azocasein at different pH ranging from 2.0 to 12.0 and the total protease activity was measured. Total protease activity in (■) IVS1, (▲) IVS2, and (●) IVS3 isolates was determined. Each value represents the average of three experiments.

**FIGURE 4:**
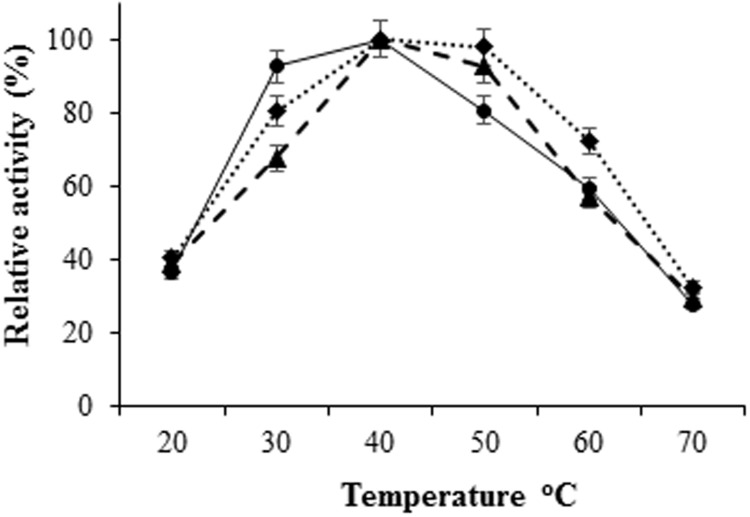
**Effect of temperature on activity of proteases from gut bacteria in *H. armigera*.** The bacterial proteases were incubated with azocasein dissolved in 0.1 M glycine-NaOH buffer at different temperatures ranging from 20 to 70°C. Total proteolytic activity was measured at 450 nm. Effect of temperature on proteases from (◆) IVS1, (●) IVS2, and (▲) IVS3 isolates was determined. Each value represents the average of three experiments.

The *H. armigera* gut bacterial protease activity was observed over the pH range 2.0–12.0, with maximum activity in alkaline environment [pH 8.0 for proteases from IVS1 (61.8 UA/mg of protein), IVS2 (39.87 UA/mg of protein), and IVS3 (50.08 UA/mg of protein)] (**Figure 3**). These proteases had a relatively low activity in the acidic pH range from 2.0 to 6.0. After pre-incubating at different temperatures, i.e., 20, 30, 40, 50, 60, and 70°C at pH 8 for 10 min, the *H. armigera* gut bacterial proteases were found to be stable at a temperature range from 30 to 50°C, with optimum activity at 40°C for proteases from IVS1 (62.37 UA/mg of protein), IVS2 (43.21 UA/mg of protein), and IVS3 (51.99 UA/mg of protein) (**Figure 4**). A rapid decrease in enzyme activity was observed above 50°C.

### Effect of Protease Inhibitors on Gut Bacterial Protease Activity

Phenylmethane sulfonyl fluoride exerted maximum inhibition of *H. armigera* gut bacterial total proteolytic activity, followed by EDTA (**Table 4**). PMSF inhibited 76, 69, and 74% of the total proteolytic activity in IVS1, IVS2, and IVS3 isolates, respectively. EDTA inhibited 58, 61, and 55% of the total proteolytic activity in IVS1, IVS2, and IVS3 isolates, respectively. Less than 50% inhibition of total proteolytic activity was seen in case of chymostatin (IVS1-41, IVS2-44, and IVS3-35%), E64 (IVS1-31, IVS2-44, and IVS3-27%), leupeptin (IVS1-27, IVS2-44, and IVS3-35%), and aprotonin (IVS1-32, IVS2-33, and IVS3-25%). Chymostatin and PMSF inhibited 88 and 85% of chymotrypsin activity in IVS1, 94 and 91% of chymotrypsin activity in IVS2 and 94 and 90% of chymotrypsin activity in IVS3.

**Table 4:** Effect of proteases inhibitors on proteases from *H. armigera* gut bacteria.

Inhibitor	Residual activity (%)					
	IVS1		IVS2		IVS3	
	Total protease	Chymotrypsin	Total protease	Chymotrypsin	Total protease	Chymotrypsin
Control	100 ± 0.0 h	100 ± 0.0 h	100 ± 0.0 f	100 ± 0.0 f	100 ± 0.0 f	100 ± 0.0 g
TPCK	54 ± 1.5 c	81 ± 2.7 e	61 ± 0.8 d	75 ± 2.0 c	65 ± 2.0 c	80 ± 0.8 d
Chymostatin	59 ± 1.5 d	11 ± 1.5 a	56 ± 1.5 c	6 ± 0.8 a	65 ± 2.5 c	6 ± 0.8 a
E64	69 ± 1.9 ef	74 ± 0.8 c	56 ± 2.3 c	100 ± 0.0 f	73 ± 1.5 d	80 ± 2.3 d
Leupeptin	73 ± 3.4 g	95 ± 2.3 g	56 ± 0.8 c	98 ± 0.8 e	65 ± 2.0 c	92 ± 1.5 f
Aprotonin	68 ± 0.8 e	83 ± 2.0 ef	67 ± 1.5 e	100 ± 0.0 f	75 ± 2.3 e	85 ± 1.5 e
EDTA	42 ± 2.8 b	77 ± 1.5 cd	39 ± 2.0 b	93 ± 1.5 d	45 ± 1.5 b	76 ± 2.0 c
PMSF	24 ± 1.9 a	15 ± 0.8 b	31 ± 0.8 a	9 ± 0.8 b	26 ± 0.8 a	10 ± 0.8 b

*The protease activity was measured in the presence of inhibitors listed. Activity in the absence of inhibitors was taken as 100% (control). Values are significant at *P* < 0.05. Values with different letters in the columns are significantly different from each other. Each value represents the average of three experiments and represented as mean ± SE.*

### Fluorescence Zymography of Bacterial Proteases

**FIGURE 5:**
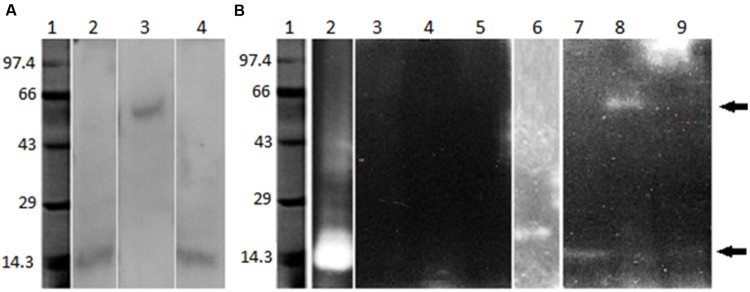
**Visualization of midgut bacterial proteases.** Sephadex G-75 column eluted bacterial protease (5 μg) were electrophoresed on 10% SDS-PAGE. After electrophoresis the gel was incubated in the trypsin or chymotrypsin assay buffer as described in text. Bacterial protease bands were visualized on **(A)** SDS-PAGE (Lane 1 – Molecular weight markers, lane 2 – proteases from IVS1, lane 3 – IVS2, lane 4 – IVS3). **(B)** Fluorescence zymography for the detection of trypsin- and chymotrypsin-like activity in the bacterial isolates (lane 1 – molecular weight markers, lane 2 – bovine trypsin, lane 3 – proteases from IVS1, lane 4 – IVS2, lane 5 – IVS3, lane 6 – bovine chymotrypsin, lane 7 – IVS1, lane 8 – IVS2 and lane 9 – IVS3. Lane 2 to 5 are for identification of trypsin proteases and 6 to 9 are for chymotrypsin proteases).

Fluorescent zymogram analysis performed for the detection of trypsin- and chymotrypsin-like proteases in gut bacterial isolates IVS1, IVS2, and IVS3. No band was observed on trypsin fluorescent zymogram, whereas, a single chymotrypsin-like band with molecular mass of ~15 kDa each in IVS1, and IVS3, and of ~65 kDa in IVS2 was visualized due to the liberated AMC (7-amino-4-methyl-coumarin) of the substrate, peptide-MCA, under UV transillumination (**Figure 5**).

### Insect Bioassay

**FIGURE 6:**
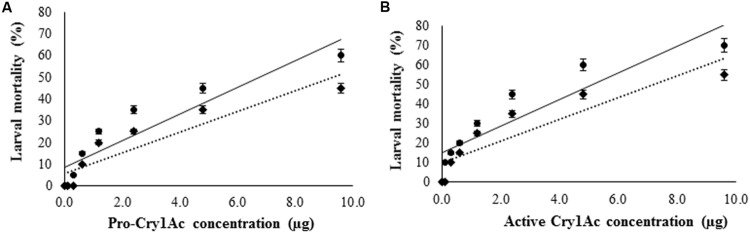
**Effect of Cry1Ac on larval survival.** The early fourth-instar larvae were used for bioassay. The larvae were reared on **(A)** pro-Cry1Ac and **(B)** active Cry1Ac (formed after the processing of pro-Cry1Ac by gut bacterial proteases) toxin treated artificial diet. (●) mortality percent in larvae reared on toxin treated artificial diet and (◆) mortality percent in larvae reared on artificial diet treated with both toxin and antibiotic cocktail. To eliminate gut bacteria the larvae were reared on diet treated with 100 μl of antibiotic cocktail (stock: 1 mg/ml of each antibiotic) per gram diet till fourth-instar, and then reared on antibiotic cocktail containing diet treated with toxin. There were three replications for each treatment and 10 larvae in each replication in a completely randomized design (CRD). Each value represents the average of three experiments.

Antibiotic cocktail was used to eliminate gut bacteria from *H. armigera*. As the concentration of antibiotic cocktail increased, the microbial colony count was decreased on nutrient agar culture plates. Among the five different concentrations tested, antibiotic cocktail at 80 and 100 μg/g diet inhibited all the gut bacteria (Supplementary Figure S2), and 100 μg of antibiotic cocktail per gram diet was further used to eliminate the gut bacteria from *H. armigera* larvae. Pro-Cry1Ac was treated with proteases from three gut bacterial isolates (IVS1, IVS2, and IVS3) and the active Cry1Ac toxin formed was used for insect bioassay. Significant increase in mortality rate was observed in larvae reared on pro-Cry1Ac treated diet [*R*^2^ = 84.96%; (LC_50_ = 5.38 μg, 95% confidence limits: lower = 2.72 and upper = 23.85)], and pro-Cry1Ac plus antibiotic cocktail treated diet [*R*^2^ = 83.70%; (LC_50_ = 9.85 μg, 95% confidence limits: lower = 4.34 and upper = 34.72)] (**Figure 6A**). Significant increase in mortality rate was observed in larvae reared on active Cry1Ac treated diet [*R*^2^ = 82.60%; (LC_50_ = 3.15 μg, 95% confidence limits: lower = 1.49 and upper = 12.86)], and active Cry1Ac plus antibiotic cocktail treated diet [*R*^2^ = 80.95%; (LC_50_ = 6.11 μg, 95% confidence limits: lower = 2.81 and upper = 44.43)] (**Figure 6B**).

**FIGURE 7:**
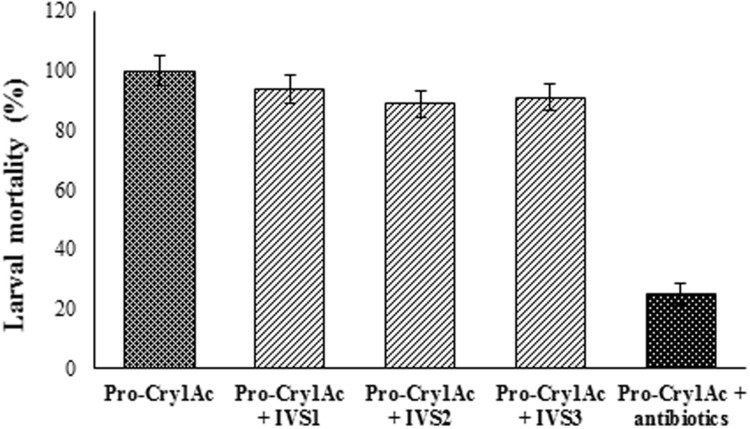
**Cry1Ac toxicity in antibiotic cocktail fed *H. armigera* larvae.** Larvae were reared until the fourth-instar on sterile artificial diet amended with 100 μl of antibiotic cocktail (Stock: 1 mg/ml of each antibiotic) per gram of diet. The antibiotic pre-treated early fourth-instar larvae were transferred to artificial diet treated with gut bacterial culture and pro-Cry1Ac (15 μg/ml of diet) and reared for 5 days. Data was recorded for Cry1Ac toxin mortality in larvae reared on artificial diet, antibiotic cocktail treated diet and gut bacteria treated diet. Equal amount of culture from IVS1, IVS2, and IVS3 isolates was added to the artificial diet. There were three replications for each treatment and 10 larvae in each replication in a CRD. Each value represents the average of three experiments.

The mortality rate triggered by Cry1Ac toxin was 100% in larvae reared on artificial diet which was reduced to 25% in the larvae reared on diet treated with antibiotic cocktail (**Figure 7**). However, in the larvae pre-treated with antibiotic cocktail when fed on diet treated with gut bacterial culture, the mortality rate triggered by Cry1Ac toxin was nearly that of larvae reared on artificial diet alone.

### Peptide Sequence Analysis

The highest peak in peptide sequence from IVS1 proteases [1374.81 Da (GLNVHPMHIGGSR)] was showing similarity with metal-dependent hydrolases from *Bacillus* sp., IVS2 proteases [1959.09 Da (IAVGGDSAGGNLAAVLSLMAR)] with alpha/beta hydrolase *Afipia massiliensis*, and IVS3 proteases [858.52 Da (RQVSASPPAT)] with serine protease pepA from *Mycobacterium tuberculosis*. The peptide sequences were subjected to protein-protein BLAST (BLAST-P) to obtain the nearest homologous neighbors, and putative conserved domains (The results for peptide sequence analysis for IVS1, IVS2, and IVS3 isolates were provided as Supplementary Figures S3–S5, respectively).

## Discussion

*Bacillus thuringiensis* toxins have been deployed on a large-scale in transgenic crops for pest management (Sharma et al., 2004; James, 2013). Large-scale cultivation of transgenic crops will exercise a strong selection pressure on the target insect pests, and continuous exposure of insect pests to *B. thuringiensis* toxins in transgenic plants will lead to development of insect resistance to transgenic crops (Carriere et al., 2010; Huang et al., 2011). The gut bacteria perform numerous functions including digestion, nutrient concentration, nitrogen fixation, and detoxification of defensive compounds (Broderick et al., 2003; Dillon and Dillon, 2004). Gut bacteria produce a wide variety of extra-cellular enzymes, including proteases. Midgut bacteria influence the insecticidal activity of *B. thuringiensis* to insect pests (Broderick et al., 2006, 2009; Paramasiva et al., 2014; Visweshwar et al., 2015). The midgut bacteria of insect host have either synergistic (Raymond et al., 2009) or protective (Broderick et al., 2006, 2009; Paramasiva et al., 2014; Visweshwar et al., 2015) effect, but the underlying mechanisms are unclear. In the present study we have isolated three isolates of protease producing gut bacteria and characterized their proteases which are involved in protoxin to toxin conversion.

Twelve dominant gut bacterial colonies were isolated from midgut of *H. armigera*, and only seven bacterial isolates exhibited proteolytic activity. Proteases from the gut bacterial isolates were purified by implying ammonium sulfate precipitation, DEAE-cellulose and Sephadex G-75 columns. Active fractions containing protease activity were pooled and incubated with the pro-Cry1Ac (130 kDa) and the liberated active toxin (65 kDa) was detected using SDS-PAGE, ELISA and Western blotting (**Figure 2**). Among the seven protease producing bacterial strains, proteases from only three strains (IVS1, IVS2, and IVS3) were able to convert inactive pro-Cry1Ac to active Cry1Ac toxin. The results suggest that the proteases produced by the midgut bacteria were involved in proteolytic processing of pro-Cry1Ac into active Cry1Ac, and thereby influencing the toxicity of *B. thuringiensis* to *H. armigera*. The Cry11Aa1 protoxin is hydrolyzed by proteases produced from bacteria (Ibarra and Federici, 1986). The *B. thuringiensis* subsp. *kurstaki* mediates endogenous proteolytic processing of 132 kDa protoxin to an active 66 kDa toxin only under denaturing/reducing conditions (Kumar and Venkateswerlu, 1997). The nucleotide homology search of IVS1, IVS2, and IVS3 isolates showed similarity with *B. thuringiensis*, *V. fischeri*, and *E. coli*, respectively. IVS2 and IVS3 isolates belong to the phylum Proteobacteria, while IVS1 isolate belongs to Firmicutes.

The optimum pH and temperature for the gut bacterial protease activity were 8 and 40°C, respectively (**Figures 3** and **4**), indicating that the proteases were alkaline in nature. Although some activity was detected at acidic pH, the maximum activity was observed at a slightly alkaline environment (pH 8.0). Gut bacterial proteases are characterized by their high activity at alkaline pH (8.0–12.0), with optimal temperature between 50 and 70°C (Al-Shehri et al., 2004). Among the seven protease inhibitors tested, the maximum inhibition of total proteolytic activity was exerted by serine protease inhibitor, PMSF followed by EDTA, indicates that the proteases were of serine type. Maximum inhibition of the protease activities was observed with chymostatin indicates that proteases were of chymotrypsin type. Fluorescence zymography of *H. armigera* gut bacterial proteases showed that the purified bacterial proteases were chymotrypsin-like, and their molecular weights were around 15 kDa for protease from IVS1 and IVS3, and 65 kDa from IVS2 isolate (**Figure 5**). In general, molecular mass of bacterial proteases ranges between 15 and 45 kDa (Kumar and Takagi, 1999; Gupta et al., 2002; Olivieri et al., 2002; Niu et al., 2006; Disney et al., 2008).

With the increased concentration of pro-Cry1Ac and active Cry1Ac the mortality rate was increased in larvae reared on artificial diet and diet treated with antibiotic cocktail. However, the reduced mortality rate was observed in the larvae reared on artificial diet treated with antibiotic cocktail compared to the larvae reared on artificial diet only (**Figure 6**). The observations are further supported from the earlier made observations that *B. thuringiensis* does not kill the larvae of the gypsy moth in the absence of indigenous midgut bacteria (Broderick et al., 2006). Elimination of the gut microbial community by oral administration of antibiotics abolished the insecticidal activity of *B. thuringiensis* in *Lymantria dispar* (L.), *Pieris rapae* (L.), and *Vanessa cardui* (L.) (Broderick et al., 2009) and in *H. armigera* (Paramasiva et al., 2014; Visweshwar et al., 2015). In antibiotic fed *H. armigera* larvae, after feeding protease producing gut bacterial culture (IVS1, IVS2, and IVS3) the mortality rate was triggered nearly that of control larvae (Larvae un-treated with antibiotics; **Figure 7**). Reduction in gypsy moth larval mortality was accompanied by reduced populations of *Enterococcus* spp. and *Enterobacter* spp. from the midguts of larvae (Broderick et al., 2006). After re-establishment of *Enterobacter* spp., the insecticidal activity was restored (Broderick et al., 2006). The mortality data was similar for active Cry1Ac produced from processing of pro-Cry1Ac by proteases purified from gut bacteria IVS1, IVS2, and IVS3.

Peptide sequence analysis from IVS1 and IVS2 showed similarity with hydrolases, and IVS3 with serine protease pepA. Serine proteases are involved in the activation of Cry protoxins through proteolytic removal of peptide fragments (Broehan et al., 2008). Several *Bacillus* species produce proteases (Beg and Gupta, 2003; Banik and Prakash, 2004; Gerze et al., 2005). Protease activity associated with whole cells of *V. fischeri* strain ES114 is a product of putative cell membrane-associated aminopeptidase (PepN; Fidopiastis et al., 2012). *E. coli* is one of the most important pathogens, and it contains numerous proteases, including metallo-, serine- and aspartic-proteases capable of catalyzing casein and some oxidized proteins (Rozkov and Enfors, 2004).

In summary, the gut bacterial proteases isolated from *H. armigera* were involved in proteolytic cleavage of pro-Cry1Ac to active toxin, indicates that proteases produced by the *H. armigera* gut bacteria influenced the proteolytic processing of protoxin to toxin, which results in insect mortality. Any changes in relative abundance and diversity of gut bacteria in insect pests will have a major bearing on development of insect resistance to *B. thuringiensis*-transgenic crops. Therefore, there is a need for understanding the role of midgut microbes in evolution of insect resistance to transgenic crops to develop strategies for deployment of transgenic crops for sustainable crop production.

## Author Contributions

HS, SK, AM, and VR designed the experiments. VR performed the experiments, analyzed the results and wrote the paper. HS, SK, and AM corrected the paper. All the authors approve the final version for publication.

## Conflict of Interest Statement

The authors declare that the research was conducted in the absence of any commercial or financial relationships that could be construed as a potential conflict of interest.

## Footnotes

1 http://www.matrixscience.com
