# Supplementary material for: Isolation and Characterization of Gut Bacterial Proteases Involved in Inducing Pathogenicity of Bacillus thuringiensis Toxin in Cotton Bollworm, Helicoverpa armigera
Source: Front Microbiol. 2016 Oct 6;7:1567. doi: 10.3389/fmicb.2016.01567 (PMC5052264; doi:10.3389/fmicb.2016.01567)
Supplement: Supplementary file 1 [file Data_Sheet_1.PDF]

Supplementary material:

Isolation and characterization of gut bacterial proteases involved in inducing pathogenicity of *Bacillus thuringiensis* toxin in cotton bollworm, *Helicoverpa armigera*

Visweshwar Regode, Sreeramulu Kuruba, Akbar SMD, and Hari Chand Sharma *

*Correspondence:
Hari Chand Sharma,
Principal Scientist - Entomology,
International Crops Research Institute for the Semi-Arid Tropics (ICRISAT),
Patancheru-502324, Telangana, India.
Email: h.sharma@cgiar.org

SUPPLEMENTARY FIGURES:

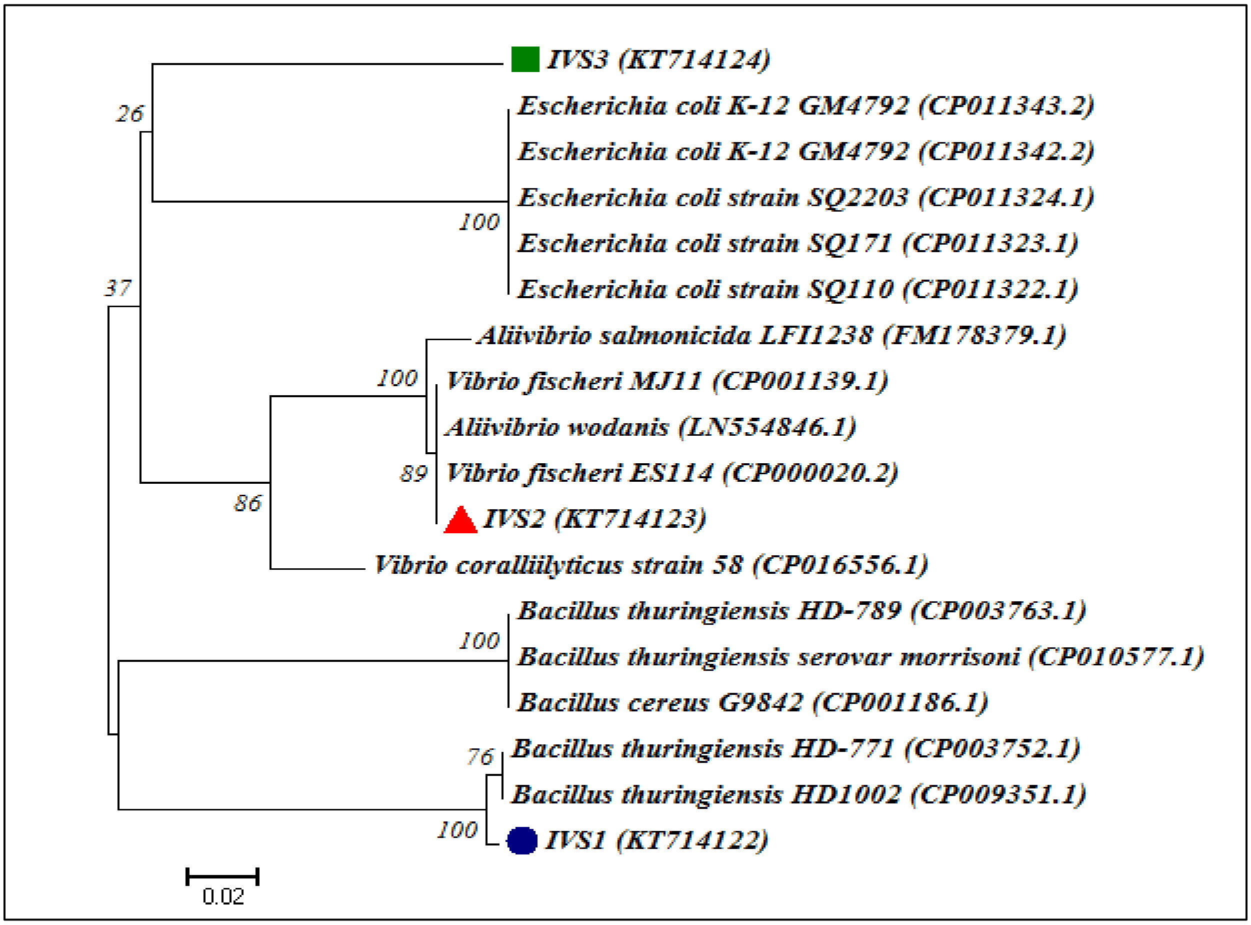

Figure S1. Phylogenetic tree for the midgut proteolytic bacterial isolates from *H. armigera*. The partial 16S rRNA gene sequences of isolates IVS1, IVS2 and IVS3 were compared with the sequences in GenBank with nucleotide-nucleotide BLAST (BLAST-N) to obtain the nearest phylogenetic neighbors (www.ncbi.nlm.nih.gov/BLAST/). The evolutionary history was inferred using the Neighbor-Joining method. The optimal tree with the sum of branch length = 0.56483209 is shown. The percentage of replicate trees in which the associated taxa clustered together in the bootstrap test (500 replicates) are shown next to the branches. The tree is drawn to scale, with branch lengths in the same units as those of the evolutionary distances used to infer the phylogenetic tree. The evolutionary distances were computed using the p-distance method and are in the units of the number of base differences per site. The analysis involved 18 nucleotide sequences. All positions containing gaps and missing data were eliminated. There were a total of 134 positions in the final dataset. Evolutionary analyses were conducted in MEGA 7.0.

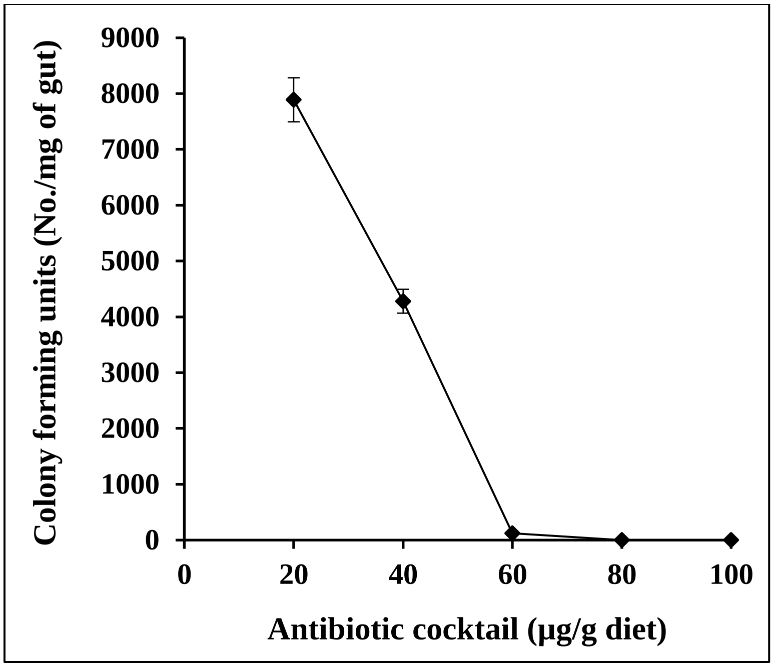

Figure S2. Elimination of bacteria from *H. armigera* midgut. The midgut bacteria were eliminated using the antibiotic cocktail described in M&Ms. The early fourth-instar larvae were used to isolate the gut bacteria. There were three replications for each antibiotic cocktail treatment and 10 larvae in each replication in a completely randomized design (CRD). The gut homogenates spread on NA media and incubated at 30 ^oC for 24 h. The microbial counts were converted into colony forming units per mg of whole gut.

```
H. armigera         ------------------------------------------------------------     0
EHA30853.1          ---------------------------mkemkvtyhghsvitvetkdhhiifdpfltgns     33
KIO55297.1          mlltrikqhkffsflkrenkktksrdevkemkvtyhghsvitvetkdhhiifdpfltgns     60
KIU13358.1          msltrikqhkffsflkrenkktkscdevkemkvtyhghsvitvetkdhhiifdpfltgns     60
WP_003245964.1      ------------------------------mkvtyhghsvitvetkdhhiifdpfltgns     30


H. armigera         --------k-advillthghndhvgdteqiak-q--------------k-glnvhpmhi     34
EHA30853.1          ltdikpedvkadvillthghndhvgdteqiakqnnalviapnelavylgwkglnvhpmhi     93
KIO55297.1          ltdikpedvkadvillthghndhvgdteqiakqnnalviapnelavylgwkglnvhpmhi     120
KIU13358.1          ltdikpedvkadvillthghndhvgdteqiakqnnalviapnelavylgwkglnvhpmhi     120
WP_003245964.1      ltdikpedvkadvillthghndhvgdteqiakqnnalviapnelavylgwkglnvhpmhi     90
                              ********************** :                 *********

H. armigera         ggsr-q----k-ltqafhgsavtdeenktitytgmpagilltvedk-tk-tifhagdtal     86
EHA30853.1          ggsrqfdfgkvkltqafhgsaitdeenktitytgmpagilltvedk----tifhagdtal     149
KIO55297.1          ggsrqfdfgkvkltqafhgsaitdeenktitytgmpagilltvedk----tifhagdtal     176
KIU13358.1          ggsrqfdfgkvkltqafhgsaitdeenktitytgmpagilltvedk----tifhagdtal     176
WP_003245964.1      ggsrqfdfgkvkltqafhgsaitdeenktitytgmpagilltvedk----tifhagdtal     146
                    ****        *********:*************************  ***********

H. armigera         fsdmk-lk-ligelnhidlaflpigdnftmgpedak-lk-laaewlr-ar-akqvvpvhy     140
EHA30853.1          fsdmk----ligelnhidlaflpigdnftmgpedak----laaewlr----akqvvpvhy     197
KIO55297.1          fsdmk----ligelnhidlaflpigdnftmgpedak----laaewlr----akqvvpvhy     224
KIU13358.1          fsdmk----ligelnhidlaflpigdnftmgpedak----laaewlr----akqvvpvhy     224
WP_003245964.1      fsdmk----ligelnhidlaflpigdnftmgpedak----laaewlr----akqvvpvhy     194
                    ******  *****************************  *********  **********

H. armigera         ntfpvieqdpeafadslpggvgk-v--------     164
EHA30853.1          ntfpvieqdpeafadslpggvgkvmsvgetiel     230
KIO55297.1          ntfpvieqdpeafadslpggvgkvmsvgetiel     257
KIU13358.1          ntfpvieqdpeafadslpggvgkvmsvgetiel     257
WP_003245964.1      ntfpvieqdpeafadslpggvgkvmsvgetiel     227
                    *********************** :
```

Figure S3. The partial amino acid sequence alignment of *H. armigera* gut bacterial proteases from IVS1. All the listed proteins are from *Bacillus subtilis.* Sequences were retrieved from Protein Data Bank (PDB) and the multiple sequence alignment was made using Clustal Omega. Matched sequences were highlighted.

*indicates identical residues in the aligned sequences.

```
H. armigera         ------------------------------------------------------------    0
EHA30853.1          ---------------------------mkemkvtyhghsvitvetkdhhiifdpfltgns    33
KIO55297.1          mlltrikqhkffsflkrenkktksrdevkemkvtyhghsvitvetkdhhiifdpfltgns    60
KIU13358.1          msltrikqhkffsflkrenkktkscdevkemkvtyhghsvitvetkdhhiifdpfltgns    60
WP_003245964.1      ------------------------------mkvtyhghsvitvetkdhhiifdpfltgns    30


H. armigera         --------k-advillthghndhvgdteqiak-q---------------k-glnvhpmhi    34
EHA30853.1          ltdikpedvkadvillthghndhvgdteqiakqnnalviapnelavylgwkglnvhpmhi    93
KIO55297.1          ltdikpedvkadvillthghndhvgdteqiakqnnalviapnelavylgwkglnvhpmhi    120
KIU13358.1          ltdikpedvkadvillthghndhvgdteqiakqnnalviapnelavylgwkglnvhpmhi    120
WP_003245964.1      ltdikpedvkadvillthghndhvgdteqiakqnnalviapnelavylgwkglnvhpmhi    90
                              ********************** :                 *********

H. armigera         ggsr-q----k-ltqafhgsavtdeenktitytgmpagilltvedk-tk-tifhagdtal    86
EHA30853.1          ggsrqfdfgkvkltqafhgsaitdeenktitytgmpagilltvedk----tifhagdtal    149
KIO55297.1          ggsrqfdfgkvkltqafhgsaitdeenktitytgmpagilltvedk----tifhagdtal    176
KIU13358.1          ggsrqfdfgkvkltqafhgsaitdeenktitytgmpagilltvedk----tifhagdtal    176
WP_003245964.1      ggsrqfdfgkvkltqafhgsaitdeenktitytgmpagilltvedk----tifhagdtal    146
                    ****        *********:************************  ***********

H. armigera         fsdmk-lk-ligelnhidlaflpigdnftmgpedak-lk-laaewlr-ar-akqvvpvhy    140
EHA30853.1          fsdmk----ligelnhidlaflpigdnftmgpedak----laaewlr----akqvvpvhy    197
KIO55297.1          fsdmk----ligelnhidlaflpigdnftmgpedak----laaewlr----akqvvpvhy    224
KIU13358.1          fsdmk----ligelnhidlaflpigdnftmgpedak----laaewlr----akqvvpvhy    224
WP_003245964.1      fsdmk----ligelnhidlaflpigdnftmgpedak----laaewlr----akqvvpvhy    194
                    ******  *****************************  *********  **********

H. armigera         ntfpvieqdpeafadslpggvgk-v--------      164
EHA30853.1          ntfpvieqdpeafadslpggvgkvmsvgetiel      230
KIO55297.1          ntfpvieqdpeafadslpggvgkvmsvgetiel      257
KIU13358.1          ntfpvieqdpeafadslpggvgkvmsvgetiel      257
WP_003245964.1      ntfpvieqdpeafadslpggvgkvmsvgetiel      227
                    *********************** :
```

Figure S4. The partial amino acid sequence alignment of *H. armigera* gut bacterial proteases from IVS2. Listed proteins are from *Variovorax paradoxes* (WP_013539303.1 and WP_047786227.1), *Polaromonas sp.,* CF 318 (WP_007864563.1) and *Neveskia soli* (WP_051748906.1). Sequences were retrieved from Protein Data Bank (PDB) and the multiple sequence alignment was made using Clustal Omega. Matched sequences were highlighted.

*indicates identical residues in the aligned sequences.

```
AMC66696.1      msqdrfadfpalpldpsamvaqvgpqvvnintklgynnavgagtgividpngvvltnnhv  60
H. armigera     ------------------------------------------------------------  0
AEJ45301.1      ------------------------------------------------------------   0


AMC66696.1      iagatdinafsvgsgqtygvdvvgydrtqdvavlqlrgagglpsaaigggvavgepvvam  120
H. armigera     ------------------------------------------------------------  0
AEJ45301.1      -----------------------------------------------mggvavgepvvam  13


AMC66696.1      gnsggqggtpravpgrvvalgqtvqasdsltgaeetlngliqfdaaiqpgdsggpvvngl  180
H. armigera     ------------------------------------------------------------  0
AEJ45301.1      gnsggqggtpravpgrvvalgqtvqasdsltgaeetlngliqfdaaiqpgdsggpvvngl  73


AMC66696.1      gqvvgmntaasdnfqlsqggqgfaipigqamaiagqirsggghppfisglppssawvlst  240
H. armigera     ------------------------------------------------------------  0
AEJ45301.1      gqvvgmntaasdnfqlsqggqdspfrsgrrwrsrarsdrvgghppfisglppssawvlst  133


AMC66696.1      ttatahesnawsgalrrqvsasppat  266
H. armigera     ----------------rqvsasppat  10
AEJ45301.1      ttatahesnawsgalrrqvsasppat  159
                                **********
```

Figure S5. The partial amino acid sequence alignment of *H. armigera* gut bacterial proteases from IVS3. All the listed proteins are from *Mycobacterium tuberculosis.* Sequences were retrieved from Protein Data Bank (PDB) and the multiple sequence alignment was made using Clustal Omega. Matched sequences were highlighted.

*indicates identical residues in the aligned sequences.
